# “Road to Rio”: A Case Study of Workload Periodization Strategy in Rugby-7s During an Olympic Season

**DOI:** 10.3389/fspor.2019.00072

**Published:** 2020-01-29

**Authors:** Julien Robineau, Bruno Marrier, Yann Le Meur, Julien Piscione, Alexis Peeters, Mathieu Lacome

**Affiliations:** ^1^Research Department, Fédération Française de Rugby, Paris, France; ^2^EA6312 Laboratoire de Motricité Humaine Expertise, Sport, Santé, Université de Toulon, Nice, France; ^3^Performance Department, Paris Saint-Germain F.C., Paris, France

**Keywords:** training load, external load, internal load, preseason, in-season, olympic games

## Abstract

The objective of this manuscript was to examine the periodization strategy of an international Rugby-7s team during an Olympic season. Training load data were collected in 14 elite male players over a 48-week period during the 2015–2016 Olympic season. The season consisted of 3 macrocycles including: preseason (12-weak duration), in-season (25-weak) fragmented into four 4–7 weeks mesocycles (In-1–4) and the final preparation for the Rio 2016 Olympic Games (Olympic preparation, 11-weak). External training load (TL) such as the total distance (TD), the high-intensity distance (HID) and the number of accelerations performed, was monitored in training and competition over the entire duration of the season using a global positioning system (GPS) devices. The rating of perceived exertion (RPE) was multiplied by the session duration (min) to provide an internal TL (session-RPE) value for all training sessions and competitions. The Olympic preparation may enable planning of higher external TL compared to the preseason (TD, 21 ± 13%, *moderate*; total accelerations, 27 ± 4%, *moderate*) whereas no difference was observed for internal TL values between these two periods. High-intensity distance (HID) and internal TL (session-RPE) were lower (−11.0 ± 7.8%, *small* and −38 ± 3%, *moderate*, respectively) during the in-season compared to preseason. Internal TL, TD as well as HID were lower in the third in-season mesocycle (In-3) compared with the first in-season mesocycle (In-1) (−25 ± 12%, *moderate*; −32 ± 4%, *moderate*; −49 ± 8%, *moderate*, respectively). The staff managed the workload considering the in-season as the main part of the “Road to Rio.” The strategy to reduce the workload at the middle of the season and to induce weeks of regeneration at the end of the in-season was highlighted by the training availability of 100% of the squad at the beginning of the Olympic preparation. The workload periodization strategy of an Olympic season differs from the strategy previously described during a non-Olympic season.

## Introduction

In elite Rugby-7s, a typical season involves a long pre-season (~12 weeks) and an in-season competitive period in which the World Rugby Sevens Series (WRSS) is played. The WRSS involves five sequences of two tournaments played over 2 consecutive weeks interspaced by preparation lasting 4–7 weeks. A substantial amount of travel to participate in the 10 tournaments is necessary, frequently over long distances. As such long-haul travel can cause jetlag (Fowler et al., 2017) and when combined with the intense demands of match-play (Ross et al., 2015; Fuller, 2018) may partly explain why the incidence and the severity of match injuries in elite Rugby-7s are significantly higher than those reported in elite Rugby XV competition (Fuller et al., 2010). A recent study on training periodization in Rugby-7s players emphasized a gradual increase in the number of unavailable players and a moderate decrease in well-being scores from the middle of the season (Marrier et al., 2018b). Research has also shown that lower body maximal power and strength values declined over the course of the season (Mitchell et al., 2016). Consequently, it seems that player well-being, recovery, and physical condition are potentially negatively affected across the WRSS.

These observations underline the importance of periodization strategies to help develop and maintain the physical fitness and well-being of Rugby-7s players throughout the competitive season. While up to now only one study has described training periodization in Rugby-7s players competing in the WRSS (Marrier et al., 2018b), results highlighted difficulties in maintaining high-load training periods throughout the season despite ~4–7 weeks of training separating each competitive block. The recent inclusion of Rugby-7s as an Olympic sport could potentially further compound these periodization issues. The Olympics are held ~11 weeks after the WRSS final tournament and as such players will probably follow different preparation and load management regimens across the season. Yet to our knowledge, no studies have described the training load (TL) undertaken by professional Rugby-7s players during an entire season with the addition of the Olympic Games. Therefore, the purpose of the present study was to examine the training periodization strategy of an international Rugby-7s team during different phases of the season: preseason, in-season and preparation for the Olympics using internal and external measures of workload. The analysis included comparison of TL data across the different seasonal periods.

## Methods

### Participants

A cohort of 14 elite athletes playing within the French national Rugby-7s team gave their written informed consent to participate (age: 26 ± 5 years, height: 179 ± 9 cm, body mass: 90 ± 11 kg). Only players participating across the entire season were selected. This protocol was conducted in accordance with the Code of Ethics of the World Medical Association (Declaration of Helsinki). The local committee waived the requirement for ethical approval for this study because all measurements were performed in the context of their national team's daily routine training monitoring (Winter and Maughan, 2009) in accordance with the national legislation and the institutional requirements.

### Design

**Figure 1:**
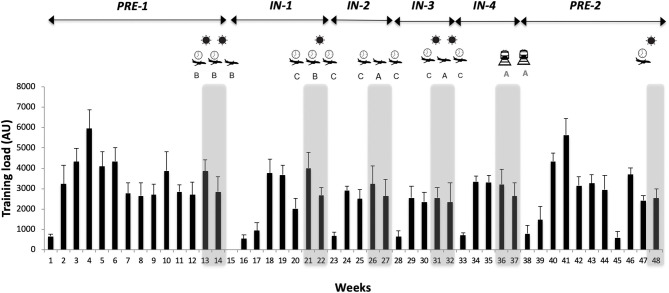
Training distribution expressed by perceived workload per week within cycle and mesocycle (mean ± SD). Gray bands represent the competition weeks and the end of each mesocycle. 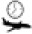: each pre and post tournament travel was classified into 3 categories (A, B and C) based on distance travel and time zones number; category-A (baseline travel): traveled ≤ 3 h and crossed ≤ 2 time zones (not expected to cause significant travel fatigue or jet lag); category-B (long-distance travel): traveled ≥ 4 h and crossed ≤ 2 time zones (likely to cause travel fatigue but not jet lag); category-C (long-distance travel across multiple time zones): traveled ≥ 10 h and crossed ≥ 6 time zones (travel fatigue and jet-lag effects may likely require more than 5 days for full recovery). 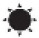: tournaments completed in “hot ambient conditions”, AU: arbitrary units.

A prospective longitudinal research design was conducted. TL data were collected over a 48-week period during the 2015–2016 Olympic season. The team competed in the WRSS participated in 10 tournaments held in different countries around the world (Figure 1).

**Figure 2:**
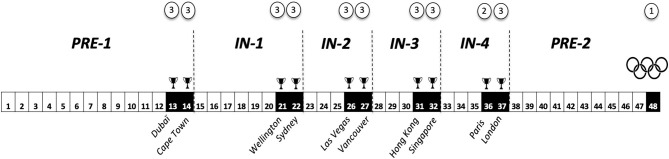
Schematic representation of the 2015–16 season. In the white circles, the number represents the tournament priority in the season: level 3 for medium importance (WRSS tournament), level 2 for high importance (WRSS tournament at home) and level 1 for maximal importance (such as Olympic Games or World Cup). Dotted bars delimit the macrocycle and mesocycle. Tournaments started at the end of the antepenultimate week of each mesocycle.

The season was broken into 3 macrocycles (Figures 1, 2) including: preseason (12-weak duration), in-season (25-weak duration) fragmented into four 4–7 weeks mesocycles (In-1–4), each punctuated by two consecutive tournaments, and the preparation period for the Rio 2016 Olympic Games (Olympic preparation, 11-weak duration).

### Methodology

#### Sampling

All team and individual training sessions were analyzed. Some data were not included in the analysis when the players did not follow the planned training program prescribed by the staff owing to illness, rehabilitation following injury, or personal reasons.

#### Training and Competition Quantification

Data regarding internal TL values were obtained using Foster's method (Foster et al., 2001). Thirty minutes after the end of each training session and match, players were asked to provide a rating of perceived exertion (RPE) using a custom-designed application installed on their smartphone. This method aimed to minimize factors that may influence a player's RPE rating, such as peer pressure and replicating other players' ratings (Haddad et al., 2017). To obtain an internal TL value for all training sessions and competitions, the rating is multiplied by exercise duration (min).

External TL was monitored in training and competition over the entire duration of the season using a global positioning system (GPS) devices. Each player wore a 16 Hz unit (Sensor Everywhere V2, Digital Simulation, Paris, France) in a lycra vest or in a bespoke pocket fitted in their playing jersey which positioned the unit on the upper thoracic spine between the scapulae. Detailed information on the present GPS devices has been provided in a previous article (Marrier et al., 2018b). External TL could not be monitored for two tournaments (C2 at In-2 and In-3). This was due to the stadium structure preventing capture of the satellite signals. For these two tournaments, the GPS data were extrapolated on the basis of the average variation of each GPS variable observed from the first to the second tournament in each competitive block.

Metrics included the total distance (TD) covered (m), the distance traveled above maximal aerobic speed (MAS) (high-intensity distance, HID) and the number of accelerations performed (>2.5 m·s^−2^ for a duration above 0.5 s). Individually determined MAS was used to individualize the high intensity speed threshold for each player. MAS values were determined during an incremental field test (“University Test of Bordeaux 2”) (Marrier et al., 2018b). This test was performed three times throughout the year and therefore, the MAS threshold was updated.

#### Time Period Analysis

Each variable was analyzed by (i) macrocycles and (ii) mesocycles to provide a full analysis of the season (Figures 1, 2).

### Statistical Analysis

Statistical analyses were performed using R statistical software (R 3.1.0, R Foundation for Statistical Computing) using the lme4 and psychometric package. Individual TL data higher than median value −2SD were retained for the analysis in order to consider only the players following the planned team training program. Data are presented as mean ± SD unless otherwise stated. Means and standard deviations were derived from the generalized linear model, with the distribution and link function contingent on the nature of the dependent variable. The normal distribution was chosen for all variables. For each analysis, the period was included as a fixed effect, while players were included as random effects. The percentage differences between mean values with 90% confidence intervals (CI) are reported. Data were analyzed using the magnitude-based inference approach (Hopkins et al., 2009). To reduce any possible bias arising from non-uniformity of error, all data were log transformed before analysis. The magnitude of the within-group changes was interpreted by using effect size (Cohen's *d*) values of the between-athlete variation as thresholds for *small* (0.2–0.6); *moderate* (0.6–1.2); *large* (1.2–2.0); *very large* (2.0–4.0) and *extremely large* (>4.0) differences (Hopkins et al., 2009). Quantitative chances of higher or lower values than the smallest worthwhile change (SWC, equal to a Cohen's *d* of 0.2) were evaluated qualitatively as follows: <1%, *almost certainly not*; 1–5%, *very unlikely*; 5–25%, *unlikely*; 25–75%, *possible*; 75–95%, *likely*; 95–99.5%, *very likely*; and >99.5%, *most likely*. In the case of having beneficial/better or detrimental/power changes were both >5% higher or lower values were 5%, the true difference was assessed as *unclear* (Hopkins et al., 2009).

## Results

### Macrocycles TL Analysis

HID and internal load were *likely* and *almost certainly* higher (11.0 ± 7.8%, *small* and 38 ± 3%, *moderate*, respectively) during the pre-season than during the in-season period (Table 1). However, *unclear* differences in TD and total number of accelerations were observed between the preseason and in-season periods (Table 1). TD and total accelerations were *likely* to *almost certainly* higher during the Olympic preparation period compared to the preseason (21 ± 13%, *moderate* and 27 ± 4%, *moderate*, respectively). An *unclear* difference was observed for HID and internal load values between these two periods (Table 1).

**Table 1:** Changes in internal and external load between macrocycles.

		**Mean ± SD**	**% Diff/PRE-1**	**ES/PRE-1**	**Qualitative effect**
s-RPE (a.u/week)	PRE-1	3,513 ± 267			
	**In-season**	**2,548 ± 216**	**−38.0 ± 3.4%**	**−0.80 ± 0.07**	***Almost certainly*** **–*****ive***
	PRE-2	3,350 ± 289	−4.6 ± 8.3%	−0.13 ± 0.23	*Possibly trivial*
TD(m/week)	PRE-1	17,881 ± 1,424			
	In-season	17,017 ± 1,361	−4.6 ± 5.7%	−0.15 ± 0.19	*Possibly trivial*
	**PRE-2**	**21,716 ± 1,889**	**21.0 ± 13.0%**	**0.7 ± 0.41**	***Very likely +ive***
HID (m/week)	PRE-1	2,013 ± 248			
	In-season	1,814 ± 249	−11.0 ± 7.8%	−0.26 ± 0.18	*Possibly* –*ive*
	PRE-2	2,212 ± 255	9.9 ± 7.9%	0.25, 0.2	*Possibly +ive*
Acc (n/week)	PRE-1	94 ± 11			
	In-season	99 ± 12	5.6 ± 6.9%	0.15 ± 0.19	*Possibly trivial*
	**PRE-2**	**120 ± 11**	**27.0 ± 4.2%**	**0.68 ± 0.10**	***Almost certainly +ive***

*SD, standard deviation; ES, Effect size ± 90%; CL, Confidence limits; –ive, negative; +ive, positive; s-RPE, rate of perceived exertion; TD, total distance; HID, high-intensity distance; Acc, acceleration; PRE-1, preseason; PRE-2, preparation to Olympics. Bold values emphasize clear differences*.

### Mesocycles TL Analysis

Overall, *unclear* differences were observed between In-1 and In-2 and In-4 for all TL variables (Table 2). Internal load, TD as well as HID were *very likely* to *almost certainly* lower in In-3 compared with In-1 (−25 ± 12%, *moderate*; −32 ± 4%, *moderate*; −49 ± 8%, *moderate*, respectively, Table 2).

**Table 2:** Changes in internal and external load between in-season cycles.

		**Mean ± SD**	**% Diff/IN-1**	**ES/IN-1**	**Qualitative effect**
s-RPE (a.u/week)	IN-1	2,606 ± 291			
	IN-2	2,446 ± 269	−6.1 ± 13.0%	−0.21 ± 0.46	*Unclear*
	**IN-3**	**1,958 ± 296**	–**25.0 ± 12.0%**	–**0.70 ± 0.33**	***Very likely*** –***ive***
	IN-4	2,730 ± 257	4.8 ± 13.0%	0.14 ± 0.37	*Unclear*
TD (m/week)	IN-1	18,999 ± 1,389			
	IN-2	17,425 ± 1,465	−8.3 ± 8.1%	−0.39 ± 0.38	*Possibly* –*ive*
	**IN-3**	**12,909 ± 1,593**	–**32.0 ± 4.2%**	–**1.10 ± 0.14**	***Almost certain*** –***ive***
	IN-4	17,527 ± 1,408	−7.8 ± 8.0%	−0.39 ± 0.40	*Possibly* –*ive*
HID (m/week)	IN-1	2,055 ± 307			
	IN-2	1,933 ± 295	−5.9 ± 14.0%	−0.14 ± 0.33	*Possibly trivial*
	**IN-3**	**1,048 ± 310**	–**49.0 ± 7.6%**	–**1.10 ± 0.17**	***Almost certain*** –***ive***
	IN-4	2,027 ± 287	−1.4 ± 13.0%	−0.04 ± 0.36	*Unclear*
Acc (n/week)	IN-1	85.8 ± 15.7			
	IN-2	**120.1 ± 15.2**	**40.0 ± 11.0%**	**0.85 ± 0.23**	***Almost certain +ive***
	IN-3	74.3 ± 15.8	−13.0 ± 16.0%	−0.29 ± 0.34	*Possibly* –*ive*
	**IN-4**	**110.2 ± 14.9**	**28.0 ± 12.0%**	**0.68 ± 0.29**	***Very likely +ive***

*SD, standard deviation; ES, Effect size ± 90%; CL, Confidence limits; –ive, negative; +ive, positive; s-RPE, rate of perceived exertion; TD, total distance; HID, high-intensity distance; Acc, acceleration; IN-1, 2, 3, and 4: In-season mesocycle 1, 2, 3, and 4. Bold values emphasize clear differences*.

## Discussion

The present study aimed to highlight the loading strategies during an Olympic season in international Rugby-7s players using internal and external TL data. The workload periodization strategy described in this article was designed and implemented by the team's coaching and sports science staff. Major findings showed clear differences in measures of internal and external TL between the macrocyles of the season. Workload was substantially reduced during the In-season and increased once again during the Olympics preparation macrocycle.

### The Preseason Phase

The longer preseason in Rugby-7s allows for implementation of a greater mean weekly internal TL (3,513 ± 267 AU, arbitrary units), including gym and field sessions, than those usually measured in other team sports that have a shorter preseason; for example Rugby Union (2,175 ± 380 AU) (Cross et al., 2016). However, in comparison to the preseason period during a non-Olympic season (Marrier et al., 2018b), the workload reported in preseason during an Olympic season was similar. Indeed, both contained four high workload weeks with internal load values superior to 4,000 AU suggesting there was no change in preparation strategies. In general, Rugby-7s practitioners implement a high TL during the preseason in order to develop a strong neuromuscular and metabolic base to respond to the demands of the sport. Another aim of the Rugby-7s preseason program is typically to try to reduce the risk of injury during the following competitive phase. Rugby players participating in a greater number of preseason sessions notably report a lower likelihood of injury throughout the competitive season (Windt et al., 2017).

Another objective of the preseason targeted by the team's staff was to analyze supercompensation kinetics regarding specific physical qualities (weeks 3–9) in order to adjust the duration of the taper phase before the Olympic tournament. The preseason period was considered to be an opportunity to anticipate the Olympic preparation cycle and identify which periodization strategy would be implemented to optimize the physical performance levels of the players during the Olympic event. These results have been detailed in a previous publication (Marrier et al., 2017). The present preseason was also used to introduce repeated sprint training in hypoxia normobaric conditions (RSH). This method is considered efficient to significantly improve mean repeated-sprint performance while an additional positive (but non-significant) effect has been reported on best repeated-sprint and maximal oxygen uptake (VO_2max_) (Brocherie et al., 2017).

### The In-season Phase

Similar to findings reported in other team sports (Rogalski et al., 2013; Malone et al., 2015; Moreira et al., 2015), both HID and internal load measured in the present study were lower during the in-season compared to in the preseason phase (11 ± 7.8 and 38 ± 3.4%, respectively). These results were likely due to a shift in training focus to ensure that players were recovered between competition blocks (Moreira et al., 2015). Coaching staff in an international Rugby-7s team must keep in mind that the Olympics are held after this competitive phase, hence there is interest to manage TL and reduce the impact of training fatigue throughout the season. Lower internal load measured during in-season in the present study may be explained by less frequent resistance and metabolic sessions.

Initially, in view of the constraints of the WRSS competition format, the present team adopted strategy was to allow “key players” or high game-time players to recover mid-season in order to preserve their physical integrity. The staff's decision to lighten the workload (2,606 ± 291 vs. 1,958 ± 296 AU; −25.0 ± 12.0%) after the third block of competition, during In-3, was motivated by three main factors: (i) to help players cope with a long-haul transmeridian travel toward the east-side which can have detrimental effects on sleep, fatigue and motivation (Fowler et al., 2017); (ii) to ensure player health and well-being as both tournaments of the third block were performed on synthetic grass which causes many skin burns and increases muscle soreness (Williams et al., 2016); (iii) to avoid the decrement of lower body maximal power values previously measured throughout the season in international Rugby-7s players (Mitchell et al., 2016).

Another temporal window identified by the staff to allow recovery in the present players with high game-time was just after the penultimate tournament, played at home so considered to be “of high importance.” Thus, the staff decided to “skip” the last tournament of the season which had no influence on the WRSS general ranking. Therefore, some players had three “regeneration” weeks before commencing preparation for the Olympics. In general, studies (Mujika and Padilla, 2003; Bosquet et al., 2007) have shown that the decrease in VO_2max_ and maximal force is significant, respectively, from the second and third week onwards of inactivity. In view of these results, the staff allowed the players only one full recovery week and two progressive “return to train” weeks with low to moderate TL (673 ± 458–1,475 ± 666 AU). The strategy to not give importance in one tournament of the WRSS makes sense during an Olympic season when the main objective takes place after the in-season. The present authors do not see any interest to apply this strategy during a non-Olympic season when the main objective focused on the WRSS ranking.

The decision to reduce the TL during an in-season mesocycle and to impose 3 weeks of regeneration at the end of the WRSS has been taken in order to optimize the players' well-being. The technical staff managed the workload considering the in-season as a main part of the “Road to Rio.” This strategy was highlighted by the training availability of 100% of the squad players at the beginning of the last phase of preparation.

### The Olympic Preparation Phase

The configuration of the season 2015/2016, with the Olympic tournament positioned 11 weeks after the end of the WRSS, provided another opportunity to implement a high workload training programme. The aim of the staff was to develop players' physical qualities targeting optimal fitness levels for the Olympics. Mean values for TL variables including TD and number of accelerations per week, were higher during the Olympic preparation compared to the preseason (21 ± 13% and 27 ± 4%, respectively) even though internal load values were identical between these two phases. The higher external load measured during the Olympic preparation, associated with a constant internal load, suggested players were fitter compared with preseason, where they were returning to play following a long period of inactivity. The higher total of accelerations per week may be explained by a specific workload orientation toward power and speed qualities development. The reduction of workload at mid-season as well as the regeneration break occurring after the last competition block would partly explain the full training availability of the players at the beginning of the final Olympic preparation.

The Olympic preparation phase was broken down into four steps. In the first step, the staff again implemented a high TL mesocycle (2 weeks), focused on RSH (2–3 sessions per week), during which a 7 day training camp was planned outside the national center. The second step was devoted to a competition block including two tournaments. The TL was reduced 1 week before the first tournament to reduce the risk of fatigue for the ensuing games. Reducing workload immediately after TL intensification and accumulation may help reduce the potential for injury and illness (Jones et al., 2017). This competitive block ended with the announcement of the list of selected players for the Olympics which might have induced emotional stress and mental fatigue (Van Cutsem et al., 2017). In this context, the staff decided to propose one low TL week performed alone by the players (1,118 ± 202 AU) just before the last high TL microcycle.

For the third step, the staff planned a simulation of the competition, consisting of 6 games, almost 3 weeks before the Olympic tournament. Overall, including the preconditioning session performed 3 h before the first simulated game (Marrier et al., 2018a), players performed seven metabolic sessions, simulating Rugby-7s games, with intensities higher than real game demands (>120 m.min^−1^). Another objective, during this last high TL microcycle, was to acclimatize the athletes to the heat and humidity observed in Rio de Janeiro. It is generally accepted that chronic exposure to heat stress enhances thermoregulatory responses, increases maximal aerobic capacity and improves thermal comfort in the heat (Daanen et al., 2018). Each training day, performed in hot weather conditions, was punctuated by passive heat exposure in sauna, hammam, and hot bath environments (Saunders et al., 2019).

Finally, the final step of the “Road to Rio” involved a 2-week tapering phase according to results of the fitness test observed during the preseason (Marrier et al., 2017). The reduction of TL began from the departure to Rio which corresponded to 14 days before the competition. The first days following the journey were dedicated to managing jet lag, recovery after long-haul travel (Mitchell et al., 2016) and return to training progressively. A final high-intensity session, in the form of opposition against another team was placed 5 days before the beginning of competition.

### Practical Applications

This case report provides information relating to the workload and periodization strategies employed by an international Rugby-7s team during an Olympic season. The results showed that: (i) the preseason, considered as a crucial window to program high workload can be used as a mean to plan and validate the final preparation to the Olympics; (ii) low to moderate TL during the in-season cycles associated with periods of regeneration in order to limit fatigue, helped commence the final preparation without any injured players; (iii) the final cycle of preparation, depending on its duration, may enable to plan higher TL thanks to improved subjective well-being of the players, and friendly games to maintain the rhythm of competition.

## Conclusions

This study is the first to describe the training periodization in Rugby-7s players competing in the WRSS during an Olympic season. This analyze emphasizes that the “Road to Rio” began long before the last final cycle of preparation. Many variables such as travel, jetlag, game-time, competition field surface and injuries have to be considered throughout the Olympic season. Complementing the players responses to TL by objective and subjective measurements aids optimization of the workload periodization strategy.

## Data Availability Statement

The datasets generated for this study are available on request to the corresponding author.

## Ethics Statement

The studies involving human participants were reviewed and approved in accordance with the Code of Ethics of the World Medical Association (Declaration of Helsinki). The patients/participants provided their written informed consent to participate in this study.

## Author Contributions

All authors listed have made a substantial, direct and intellectual contribution to the work, and approved it for publication.

### Conflict of Interest

ML was employed by the company Paris Saint-Germain F.C. The remaining authors declare that the research was conducted in the absence of any commercial or financial relationships that could be construed as a potential conflict of interest.

## References

[B1] Bosquet L. Montpetit J. Arvisais D. Mujika I. (2007). Effects of tapering on performance: a meta-analysis. Med. Sci. Sports Exerc. 39, 1358–1365. 10.1249/mss.0b013e31806010e0 17762369

[B2] Brocherie F. Girard O. Faiss R. Millet G. P. (2017). Effects of repeated-sprint training in hypoxia on sea-level performance: a meta-analysis. Sports Med. 47, 1651–1660. 10.1007/s40279-017-0685-3 28194720

[B3] Cross M. Williams S. Trewartha G. Kemp S. Stokes K. (2016). The influence of in-season training loads on injury risk in professional rugby union. Int. J. Sports Physiol. Perform. 11, 350–355. 10.1123/ijspp.2015-0187 26309331

[B4] Daanen H. A. M. Racinais S. Périard J. D. (2018). Heat acclimation decay and re-induction: a systematic review and meta-analysis. Sports Med. 48, 409–430. 10.1007/s40279-017-0808-x 29129022 PMC5775394

[B5] Foster C. Florhaug J. A. Franklin J. Gottschall L. Hrovatin L. A. Parker S.. (2001). A new approach to monitoring exercise training. J. Strength Cond. Res. 15, 109–115. 10.1519/00124278-200102000-00019 11708692

[B6] Fowler P. M. Murray A. Farooq A. Lumley N. Taylor L. (2017). Subjective and objective responses to two Rugby 7's world series competitions. J. Strength Cond. Res. 33, 1043–1055. 10.1519/JSC.0000000000002276 29016478

[B7] Fuller C. W. (2018). Modelling injury-burden in rugby sevens. J. Sci. Med. Sport. 21, 553–557. 10.1016/j.jsams.2017.10.019 29097229

[B8] Fuller C. W. Taylor A. Molloy M. G. (2010). Epidemiological study of injuries in international Rugby Sevens. Clin. J. Sport Med. 20, 179–184. 10.1097/JSM.0b013e3181df1eea 20445357

[B9] Haddad M. Stylianides G. Djaoui L. Dellal A. Chamari K. (2017). Session-RPE method for training load monitoring: validity, ecological usefulness, and influencing factors. Front. Neurosci. 11:612. 10.3389/fnins.2017.00612 29163016 PMC5673663

[B10] Hopkins W. G. Marshall S. W. Batterham A. M. Hanin J. (2009). Progressive statistics for studies in sports medicine and exercise science. Med. Sci. Sports Exer. 41, 3–13. 10.1249/MSS.0b013e31818cb278 19092709

[B11] Jones C. M. Griffiths P. C. Mellalieu S. D. (2017). Training load and fatigue marker associations with injury and illness: a systematic review of longitudinal studies. Sports Med. 47, 943–974. 10.1007/s40279-016-0619-5 27677917 PMC5394138

[B12] Malone J. J. Di Michele R. Morgans R. Burgess D. Morton J. P. Drust B. (2015). Seasonal training-load quantification in elite English premier league soccer players. Int. J. Sports Physiol. Perform. 10, 489–497. 10.1123/ijspp.2014-0352 25393111

[B13] Marrier B. Durguerian A. Robineau J. Chennaoui M. Sauvet F. Servonnet A. (2018a). Preconditioning strategy in Rugby-7s players: beneficial or detrimental? Int. J. Sports Physiol. Perform. 20, 1–26. 10.1123/ijspp.2018-0505 30569798

[B14] Marrier B. Le Meur Y. Leduc C. Piscione J. Lacome M. Igarza G. (2018b). Training periodization over an elite Rugby Sevens season: from theory to practice. Int. J. Sports Physiol. Perform. 18, 1–9. 10.1123/ijspp.2017-0839 29952634

[B15] Marrier B. Robineau J. Piscione J. Lacome M. Peeters A. Hausswirth C.. (2017). Supercompensation kinetics of physical qualities during a taper in team sport athletes. Int. J. Sports Physiol. Perform. 12, 1–24. 10.1123/ijspp.2016-0607 28121198

[B16] Mitchell J. A. Pumpa K. L. Williams K. J. Pyne D. B. (2016). Variable changes in body composition, strength and lower-body power during an international rugby sevens season. J. Strength Cond. Res. 30, 1127–1136. 10.1519/JSC.0000000000001188 27003455

[B17] Moreira A. Bilsborough J. C. Sullivan C. J. Ciancosi M. Aoki M. S. Coutts A. J. (2015). Training periodization of professional Australian football players during an entire Australian Football League season. Int. J. Sports Physiol. Perform. 10, 566–571. 10.1123/ijspp.2014-0326 25405365

[B18] Mujika I. Padilla S. (2003). Scientific bases for precompetition tapering strategies. Med. Sci. Sports Exerc. 35, 1182–1187. 10.1249/01.MSS.0000074448.73931.11 12840640

[B19] Rogalski B. Dawson B. Heasman J. Gabbett T. J. (2013). Training and game loads and injury risk in elite Australian footballers. J. Sci. Med. Sport. 16, 499–503. 10.1016/j.jsams.2012.12.004 23333045

[B20] Ross A. Gill N. Cronin J. Malcata R. (2015). The relationship between physical characteristics and match performance in rugby sevens. Eur. J. Sport Sci. 15, 565–571. 10.1080/17461391.2015.1029983 25868066

[B21] Saunders P. U. Garvican-Lewis L. A. Chapman R. F. Périard J. D. (2019). Special environments: altitude and heat. Int. J. Sport Nutr. Exerc. Metab. 29, 210–219. 10.1123/ijsnem.2018-0256 30676138

[B22] Van Cutsem J. Marcora S. De Pauw K. Bailey S. Meeusen R. Roelands B. (2017). The effects of mental fatigue on physical performance: a systematic review. Sports Med. 47, 1569–1588. 10.1007/s40279-016-0672-0 28044281

[B23] Williams S. Trewartha G. Kemp S. P. Michell R. Stokes K. A. (2016). The influence of an artificial playing surface on injury risk and perceptions of muscle soreness in elite rugby union. Scand J. Med. Sci. Sports. 26, 101–108. 10.1111/sms.12402 25644277

[B24] Windt J. Gabbett T. J. Ferris D. Khan K. M. (2017). Training load injury paradox: is greater participation associated with lower in-season injuryrisk in elite rugby league players? Br. J. Sports Med. 51, 645–650. 10.1136/bjsports-2016-095973 27075963

[B25] Winter E. M. Maughan R. J. (2009). Requirements for ethics approvals. J. Sports Sci. 27:985. 10.1080/02640410903178344 19847681

